# Opioid-free anesthesia versus opioid-inclusive anesthesia for breast cancer surgery: a retrospective study

**DOI:** 10.1186/s44158-021-00008-5

**Published:** 2021-10-09

**Authors:** Pia Di Benedetto, Massimiliano Pelli, Chiara Loffredo, Rosaria La Regina, Federico Policastro, Silvia Fiorelli, Roberto Alberto De Blasi, Flaminia Coluzzi, Monica Rocco

**Affiliations:** 1 Anesthesia and Intensive Care Medicine, Department of Clinical and Surgical Translational Medicine, Sant’Andrea Hospital, Sapienza University of Rome, Via di Grottarossa 1035, 00189 Rome, Italy; 2 Department of Medical and Surgical Sciences and Biotechnologies, Sapienza University of Rome, Polo Pontino Latina, Italy

**Keywords:** Opioid-free anesthesia, Breast surgery, Ketamine, Magnesium, Postoperative pain

## Abstract

**Background:**

Breast cancer surgery is usually managed using opioid-inclusive anesthesia (OIA), although opioids are associated with several adverse events, including nausea, vomiting, and constipation. Multimodal opioid-free anesthesia (OFA) has been introduced to reduce the incidence of these side effects. In this single-center retrospective study, we investigated whether ketamine, combined with magnesium and clonidine, could effectively control postoperative pain in patients undergoing quadrantectomy, while reducing postoperative nausea and vomiting (PONV).

**Results:**

A total of 89 patients submitted to quadrantectomy were included and divided into an OFA group (38 patients) and an OIA group (51 patients) according to the received anesthetic technique. Analgesia in the OIA group was based on an intraoperative infusion of remifentanil, and analgesia in the OFA consisted of an intraoperative infusion of ketamine and magnesium sulfate. Postoperative pain in both groups was managed with nonsteroidal anti-inflammatory drugs (NSAIDs) and paracetamol. Postoperative pain, assessed with the numeric rating scale (NRS), requirements for additional analgesics, the incidence of PONV, and patient satisfaction evaluated using a QoR-40 questionnaire were compared between the two groups. Levels of pain at 30 min and 6, 12, and 24 h after surgery; number of paracetamol rescue doses; and the incidence of PONV were lower in the OFA group (*p* <0.05). Patient satisfaction was comparable in the two groups.

**Conclusions:**

A combination of ketamine, magnesium, and clonidine could be more effective than opioid-based analgesia in reducing postoperative pain and lowering PONV occurrence after quadrantectomy for breast cancer.

## Background

Breast cancer is the most commonly diagnosed neoplasm in women and accounts for nearly a third (29%) of all malignancies, representing the leading cause of cancer death among women worldwide [9]. Quadrantectomy and lumpectomy are the most frequently used techniques for breast tumor removal [42]. Because of their minimal invasiveness, they have become the choice methods for breast cancer treatment whenever possible [11].

Acute postoperative pain after breast surgery stems both from direct damage to peripheral nerve fibers and from changes in neuro-endocrine profiles [10]. However, a significant percentage of patients (20–65%) also experience chronic postoperative pain (CPOP), which is closely associated with perceived disability long after recovery from the surgical insult and greatly impacts the quality of life [21, 24, 26].

Breast cancer surgery is typically carried out using either balanced anesthesia or total intravenous anesthesia, which involves the administration of intraoperative opioids (opioid-inclusive anesthesia, OIA), either in boluses or by target-controlled infusion. Opioids’ intraoperative administration is burdened by side effects such as acute tolerance and postoperative paradoxical hyperalgesia [2]. The latter is caused by neuronal changes involving both the central and peripheral nervous systems, which result in sensitization of pain pathway s[25], and an increase in acute postoperative pain [1, 20].

The most commonly reported side effects of opioids are postoperative nausea and vomiting (PONV) and constipation [31, 38]. PONV represents the prevailing complication after breast cancer surgery with a reported incidence of 80% [44]. PONV can negatively affect well-being and patient satisfaction, increase morbidity (dehydration, wound dehiscence, pain, and immobility), and rise length of hospital stay and hospital costs [28, 44]. Additionally, it has also been hypothesized that the use of opioids may increase the risk of metastasis and cancer recurrence [8, 12].

Two anesthesia techniques, opioid-free anesthesia (OFA) and opioid-sparing anesthesia, have been introduced in order to avoid the adverse effects of opioids. Both techniques have been proven to be effective in decreasing opioid consumption in acute postoperative pain management [36]. In OFA, the main pain pathways, both central and peripheral, are blocked by drugs acting on sodium channels, G-protein-coupled receptors, and *N*-methyl-d-aspartic acid (NMDA) receptors, some of which also have anti-inflammatory activity [18]. Analgesic and adjuvant molecules used in OFA include alpha-2 receptor agonists and lidocaine [38], all of which act both on their own and synergistically with NMDA receptor antagonists, such as ketamine and magnesium sulfate, for acute and chronic pain control [41].

We hypothesized that OFA, using a combination of ketamine, magnesium, and clonidine, might provide better postoperative pain relief than OIA for patients submitted to quadrantectomy. The main aim of this retrospective study was to determine whether pain reported through a numerical rating scale (NRS) in the 24 h following surgery was lower in a group of patients receiving OFA than in a group of patients receiving OIA. Secondary objectives included evaluating differences in the incidence of PONV, the number of doses of rescue analgesics needed, and perception of the quality of care during the hospital stay until final discharge using a QoR-40 questionnaire.

## Methods

This retrospective study was approved by the Bioethics Committee of Sapienza University of Rome (no. 7028_2020). The patients involved in the study underwent quadrantectomy, without axillary lymph nodes dissection, from October 1, 2017, to March 30, 2018.

Data were extracted from anesthesiologic and nursing reports. The quality of life was evaluated using a QoR-40 questionnaire, which is routinely completed by all inpatients. Pain scores, assessed using NRS, were entered in anesthesiologic and nursing reports immediately after surgery, during the stay in the post-anesthesia care unit (PACU), and during hospitalization in the assigned ward. The NRS is an 11-point scale for self-reporting of pain where 0 = no pain and 10 = extreme pain/worst possible pain. Pain assessments were recorded at 30 min and 6, 12, and 24 h after surgery. The total use of analgesics after surgery, the need for “rescue doses,” the occurrence of PONV, and hemodynamic or respiratory instability were also recorded, both immediately after surgery and during the hospital stay.

Incomplete or incorrect records (i.e., those that did not include NRS pain scores at the correct times or did not include a correctly completed QoR-40 questionnaire) were excluded from the study. The exclusion criteria were the history of opioid, alcohol, or drug abuse; chronic pain; the use of analgesics before surgery; and psychiatric illness.

This anesthesia department has a group of anesthetists dedicated to breast surgery. One of the anesthetists regularly uses OFA, whilst the rest of the group uses OIA. The patients were divided into two groups based on the employed method of anesthesia. OFA and OIA were both administered by target-controlled infusion, following the protocols shown in Table 1.

**Table 1:** Drugs used in the anesthesia regimens of opioid-inclusive (OIA) and opioid-free (OFA) groups

OIA	OFA
**Premedication:** *Midazolam* (2 mg IV)	**Premedication:** *Midazolam* (2 mg IV) + *Clonidine* (1–2 mcg/kg IV)
**Induction:** *Propofol* (2 mg/kg IV) + *Rocuronium Bromide* (0.6 mg/kg IV) + *Remifentanil* (TCI with an effector site concentration (Cet) of 3–4 ng/ml). Orotracheal intubation.	**Induction:** *Propofol* (2 mg/kg IV) + *Rocuronium Bromide* (0.6 mg/kg IV) + *MgSO*_*4*_ (1 g IV) + *Ketamine* (0.2–0.4 mg/kg/h IV). Orotracheal intubation.
**Maintenance:** *Desflurane* or *Propofol* TCI (Schnider protocol [39] with a Cet of 4–5 mcg/ml) + *Remifentanil* TCI (Cet of 3–4 ng/ml).	**Maintenance:** *Desflurane* or *Propofol* TCI (Schnider [39] protocol with a Cet of 4–5 mcg/ml) + *Ketamine* 0.2–0.4 mg/kg/h + *MgSO*_*4*_ 8–10 mg/kg/h.

Abbreviations: *OFA* opioid-free anesthesia, *OIA* opioid-inclusive anesthesia, *TCI* target controlled infusion, *Cet* target effect-site concentration

Remifentanil infusion was stopped 30 min before the end of the surgery, then morphine 0.05mg/kg and ondansetron 4 mg were administered in the OIA group. Anesthesia reports were completed as usual during each surgery, with non-invasive blood pressure readings, heart rate, and peripheral capillary oxygen saturation (%) recorded every 5 min, from the start until emergence.

Postoperative analgesia was based on a 24-h intravenous continuous infusion of ketorolac (60–90 mg, depending on the patient’s clinical status) and metoclopramide (10 mg), using an elastomeric pump. A rescue dose of intravenous paracetamol (1 g) was administered for pain with an NRS score ≥ 5.

After emergence from anesthesia, the patients were monitored in the PACU for at least 30 min; hemodynamic parameters, respiratory function, and postoperative pain values were fully assessed and recorded on a specific chart.

Once the patients were discharged from the PACU to the assigned ward, pain was re-assessed using the NRS and the values were recorded, together with the presence or absence of PONV, in specific nursing reports. The nursing staff was also able to record hemodynamic, respiratory, and other clinically significant events, such as postoperative delirium (POD) episodes and postoperative cognitive dysfunction (POCD). PONV was treated with intravenous ondansetron (4 mg) in all patients without a history of intolerance to the drug.

Immediately before being discharged from the hospital, all patients completed another QoR-40 questionnaire. Basing on the individual score difference of questionnaires, the patients were divided into three different groups: ≤10 (group A), 10–30 (group B), and >30 (group C). Only the QoR-40 score that differed by more than 10 points from baseline values (in either a positive or negative direction) was considered as a significative change.

Descriptive analysis was performed using percentages of binary variables, average and median values for continuous variables, and by calculating their respective dispersion values. Normality was tested using the Kolmogorov-Smirnov test. An ANOVA test was used for repeated measures to evaluate postoperative pain values reported through the NRS at the chosen time intervals (30 min and 6, 12, and 24 h after surgery). A statistical comparison of postoperative pain at different time points between groups was made using Welch’s adjusted *T* test for unequal variances. PONV was considered to be a dichotomous variable (presence/absence of PONV episodes) and studied using Yates’s chi-squared test. The total administration of rescue therapy was analyzed using the Mann-Whitney *U* test for independent samples. The QoR-40 questionnaire was analyzed in qualitative terms, without a quantitative measure of variations. Data are presented as average with standard deviation or median values with a confidence interval of 95%. *P* values <0.05 were considered to be statistically significant. Data were analyzed using the software program MedCalc, version 19.0.7

## Results

**Fig. 1:**
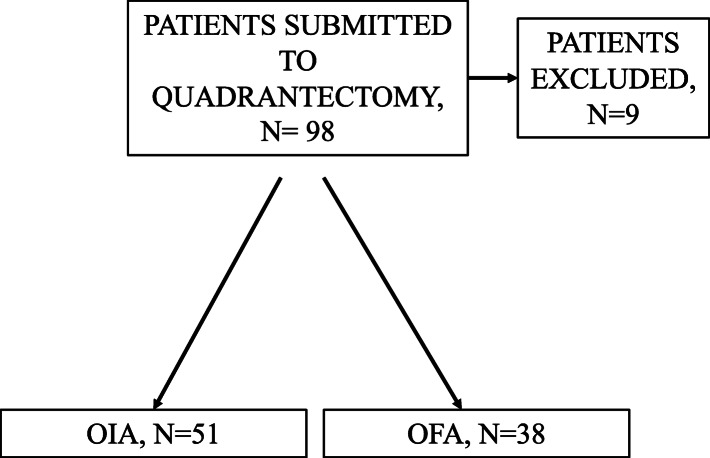
Flow diagram of the study. *Abbreviations: OFA opioid-free anesthesia, OIA opioid-inclusive anesthesia*

*N*=98 patients were submitted to quadrantectomy without axillary lymph nodes dissection during the period study, as shown in Fig. 1. After records review, four patients were excluded because they did not include a correctly completed QoR-40 questionnaire (*n*=4), history of chronic pain (*n*=2), and analgesic use before surgery (*n*=3). Patients were divided into two groups based on the method of anesthesia. The OFA group contained 38 patients, and the OIA group contained 51 patients. There were no statistically significant differences in the demographic or clinical data between the two groups, as illustrated in Table 2.

**Table 2:** Demographic features of OFA and OIA group patients

	OFA (*N*= 38)	OIA (*N*= 51)	***p*** value
**Age, years** (mean ± SD)	59.36 ± 10.16	56.40 ± 7.60	0.119
**BMI** (kg/m^2^, mean ± SD)	26.23 ± 4.79	25.04 ± 6.09	0.364
**Duration of surgery, min** (mean ± SD)	85.36 ± 19.34	89.63 ± 25.52	0.403
**Hypertension,** yes/no	7/31	10/41	0.888
**Diabetes mellitus type II,** yes/no	4/34	6/45	0.854
**ASA score,** I/II	25/13	40/11	0.276

NOTE. The data are expressed as mean ± SD or N° of patients

Abbreviations: *ASA* American Society of Anesthesiologists, *BMI* body mass index, *SD* standard deviation, *OFA* opioid-free anesthesia, *OIA* opioid-inclusive anesthesia

In both groups, adequate depth of anesthesia was achieved and maintained throughout surgery. Intraoperative hemodynamic stability was maintained in both groups during surgery and no vasoactive drugs were required.

**Fig. 2:**
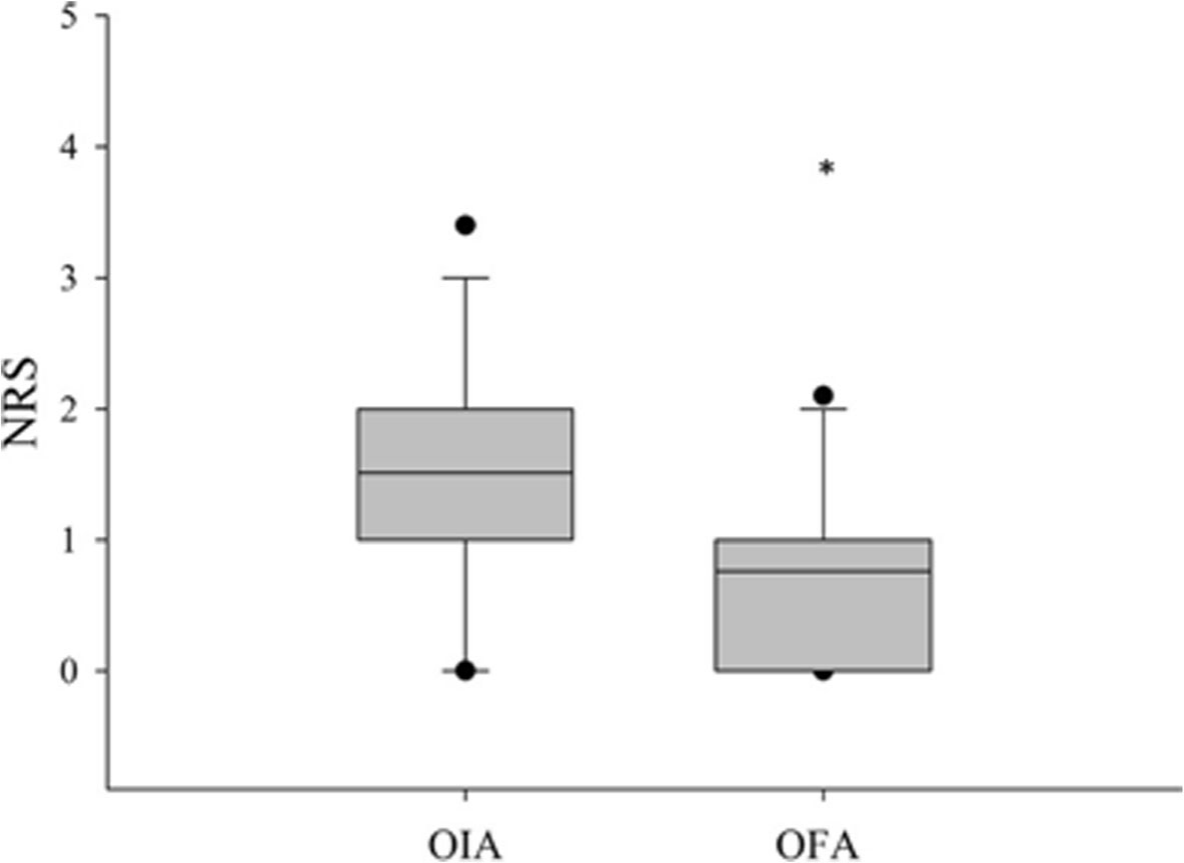
Postoperative NRS values for the OIA group and OFA group. The boundary of the box closest to 0 indicates the 25th percentile, the line within the box marks the median, and the boundary of the box furthest from 0 indicates the 75th percentile. Whiskers above and below the box indicate the 90th and 10th percentiles.

Lower NRS scores and PONV incidence were reported in the OFA group compared with the OIA group, as shown in Table 3. Visual representation of NRS pain scores is provided in Fig. 2.

**Table 3:** Pain scores at 30 min and 6, 12, and 24 h after surgery, and postoperative nausea and vomiting (PONV) incidence in the opioid-free (OFA) and opioid-inclusive (OIA) anesthesia groups

	OFA	OIA	***p*** value
**NRS 30 min**	4.28 ± 0.28	6.13 ± 0.28	**<0.0001**
**NRS 6 h**	2.94 ± 0.24	4.31 ± 0.23	**0.0001**
**NRS 12 h**	2.0 ± 0.14	3.13 ± 0.23	**0.0001**
**NRS 24 h**	1.28 ± 0.11	1.92 ± 0.15	**0.001**
**PONV (*n*/%)**	5 /13.15	19/37.25	**0.021**

The data is expressed as mean ± SD or N° of patients. *p value by *t* test or chi-squares, as appropriate;

Abbreviations: *NRS* numerical rating score, *OFA* opioid-free anesthesia, *OIA* opioid-inclusive anesthesia, *PONV* postoperative nausea and vomiting

The OIA group needed the highest number of paracetamol rescue doses, 77 in total, with a median of 2.0 administrations (CI 1–2). The OFA group required only 29 rescue doses in total, with a median of 1.0 (CI 0–1). The difference was statistically significant (*p*= 0.006).

A comparison of QoR-40 questionnaire scores between admission and discharge showed that 87% of patients, in both the OFA and OIA groups, were in subgroup A (showing a difference of ≤10 points). The vast majority of patients in both groups had no significant change in QoR-40 score during their hospital stay, suggesting no difference in perception of hospitalization quality between the OFA group and the OIA group. It is worth noting that five patients in both the OIA and OFA groups had a change in QoR-40 score of 10–30 points (subgroup B), whereas two patients in the OIA group and no patients in the OFA group had a difference greater than 30 points (subgroup C).

Either group reported any adverse reactions to drugs, and no patient required any analgesics other than paracetamol as rescue therapy.

Any patient experienced no POD or POCD, and no major cardiac, respiratory, or hemodynamic events were reported.

## Discussion

This study showed that the OFA regimen, based on continuous intravenous infusion of ketamine and magnesium, significantly reduced postoperative pain and PONV than the OIA regimen. OFA is possible by the intravenous administration of nonopioid drugs that block surgical stress and sympathetic reactions.

Only qualitative analysis was carried out on the data gathered through the QoR-40 questionnaire since 24 h was not viewed as a long enough time to speculate about reasons for improvement or worsening of the quality of life. Nevertheless, in both groups, 87% of patients showed changes of ± 10 points in QoR-40 scores at discharge from the hospital, showing that an opioid-free treatment did not worsen patients’ overall satisfaction or comfort when compared with an opioid-inclusive approach.

Although adding to OFA a locoregional block can be a valuable choice during thoracoabdominal surgeries, OFA can be very effective by combining several medications without a locoregional analgesic technique [32].

Few studies evaluated OFA technique in breast cancer surgery. In agreement with the results of the presenting study, an OFA approach demonstrated favorable results and appears to be associated with the reduction of postoperative pain scores, opioid consumption, and PONV [16, 22, 34, 43].

Studies in other surgeries also showed that OFA may result in reduced postoperative opioid requirements, as well as decreased PONV incidence, hospital length of stay, and risk for intraoperative complications [4, 17, 46].

However, in a recent randomized trial in gynecological laparoscopy, OFA demonstrated to be feasible and easy to perform, but no obvious advantages were provided in this setting over OIA with regard to PONV, pain scores, and morphine consumption [29].

A recent meta-analysis, including 33 RCTs and 2209 participants, showed no clinically significant benefits in terms of pain and opioid use after surgery, but a clear benefit with respect to a reduction in PONV was demonstrated when intraoperative opioids were avoided [37]. In a previous meta-analysis and systematic review of 23 trials, Frauenknecht et al. reported that OFA did not reduce pain scores after surgery, but it was associated with a decreased rate of PONV with a risk ratio of 0.77 (95%CI (0.61–0.97), *p* = 0.03) [14]. However, the main limitation of these metanalyses is that no precise definition of “opioid-free” was provided, and the analysis included a large miscellany of OFA techniques.

NMDA receptors have a central role in the mechanisms of OFA. These ionotropic, calcium-permeable, and receptors are activated by both glutamate and voltage changes and are involved in the transmission and modulation of pain. They also contribute to central sensitization and a phenomenon known as “wind-up,” both of which play a role in chronic pain mechanisms. Repeated stimulation of spinal C-fibers increases the spontaneous activity of NMDA receptors [35] and leads to a progressive increase in the action potential discharge’s magnitude and duration and to persistent changes in neuronal excitability [3, 7].

Many studies have shown that ketamine is effective as an analgesic both during and after different types of surgery, including abdominal, thoracic, orthopedic, and bariatric surgery. Ketamine acts as an NMDA receptor antagonist, both centrally and peripherally and is also used as an adjuvant in patient-controlled analgesia to ease PONV and to prevent CPOP [23, 46].

Several meta-analyses and randomized controlled trials have shown that magnesium sulfate, another drug commonly used in OFA, is very effective in reducing acute postoperative pain [6, 27]. Magnesium directly blocks NMDA receptors and calcium channels at rest and during depolarization, and it inhibits the release of catecholamine from the adrenal glands. Magnesium has also anti-inflammatory activity, as demonstrated by its ability to reduce levels of IL-6 and TNF [3]. NMDA receptors are blocked by ketamine, and since the binding site is in the internal portion of the channel, only open channels are blocked. All of these effects were thoroughly documented in the APMSE4 study [40], which showed that administration of ketamine and magnesium, either as a single shot or as a continuous intravenous infusion, helped to maintain good intraoperative hemodynamic stability [13]. De Oliveira et al. [5] assessed magnesium’s role, before and throughout breast surgery, as an adjuvant to continuous intravenous infusion of remifentanil. Better intraoperative hemodynamic stability and pain control after surgery were observed when compared with placebo. Administration of both ketamine and magnesium sulfate not only blocks pain transmission and its chronicization but also effectively impedes the ensuing inflammatory response.

Our results agree with those of Laskowski et al. [23] which clearly showed that ketamine-based anesthesia provided superior pain management compared with OIA. Opioid-based totally intravenous anesthesia with target-controlled infusion is linked to acute opioid tolerance because of the activation of NMDA receptors in the medulla’s dorsal horns, contemporaneous inactivation of μ-opioid receptors, increased release of dynorphines, and activation of cAMP pathways [25]. Acute tolerance to opioids increases the intensity of acute postsurgical pain.

The incidence of PONV was reduced in patients who received OFA. Complications that might arise from PONV include inhalation of gastric contents, bleeding, surgical wound re-opening, dehydration, electrolyte imbalance, and a general delay in healing and discharge from the hospital [19, 30]. The reported incidence of PONV ranges from 35% [15] to 80% [45]. Wesmiller et al. [45] showed that patients with higher levels of pain also experience an increased incidence of PONV, probably caused by larger quantities of intraoperative opioids. PONV is closely associated with receptors and transporters in the chemoreceptor trigger zone for emesis, commonly known as the area postrema, where serotonin, dopamine, catecholamine, acetylcholine, histamine, and opioids exert their effects. According to these results, we found that patients in the OIA group had both the highest postoperative pain levels and the highest incidence of PONV. The lower incidence of PONV in the OFA group could also be related to the effects of clonidine. As previously reported by Oddby-Muhrbeck et al. [33], surgical stimulation and anesthetic drugs are associated with increased vasopressin and epinephrine release. Clonidine is known to reduce epinephrine levels, explaining the lower incidence of PONV in the OFA group.

Principal limitations to our study are its monocentric and retrospective nature, its relatively small sample size, and the restriction of re-evaluation time to 24 h after surgery. In addition, the bispectral Index (BIS) was not performed during anesthesia. Such a short follow-up time made it impossible to effectively assess the effect of either method of anesthesia on the patients’ quality of life in the long term. Lack of quantitative measures for PONV, POCD, and POD and the non-application of a risk score for PONV (i.e., Apfel score) further limited our investigation since these conditions could be scored only as either present or absent. Lastly, a regional anesthetic technique such as paravertebral block or pectoral nerves block could have been considered for additional pain relief and OFA regimen implementation.

## Conclusion

In conclusion, this retrospective analysis of two different anesthesia methods, one with opioids (OIA) and one without opioids (OFA), showed that an opioid-free approach based on concomitant administration of ketamine and magnesium as a continuous intravenous infusion is safe and effective. OFA was associated with reduced postoperative pain in the first 24 h after surgery, lower need for additional analgesic drugs, and reduced incidence of PONV. The patients’ comfort levels were comparable to those of patients receiving OIA.

## Data Availability

The data that support the findings of this study are available upon reasonable request.

## Abbreviations

NRS: numerical rating score
OFA: opioid-free anesthesia
OIA: opioid-inclusive anesthesia

## References

[1] Al-Hasani R, Bruchas MR (2011). Molecular mechanisms of opioid receptor-dependent signaling and behavior. Anesthesiology.

[2] Angst MS, Clark JD (2006). Opioid-induced hyperalgesia: a qualitative systematic review. Anesthesiology.

[3] Aryana P, Rajaei S, Bagheri A, Karimi F, Dabbagh A (2014). Acute effect of intravenous administration of magnesium sulfate on serum levels of interleukin-6 and tumor necrosis factor-α in patients undergoing elective coronary bypass graft with cardiopulmonary bypass. Anesthesiol pain Med.

[4] Bakan M, Umutoglu T, Topuz U, Uysal H, Bayram M, Kadioglu H, Salihoglu Z (2015). Opioid-free total intravenous anesthesia with propofol, dexmedetomidine and lidocaine infusions for laparoscopic cholecystectomy: a prospective, randomized, double-blinded study. Brazilian J Anesthesiol.

[5] De Oliveira GS, Bialek J, Fitzgerald P (2013). Systemic magnesium to improve quality of post-surgical recovery in outpatient segmental mastectomy: a randomized, double-blind, placebo-controlled trial. Magnes Res.

[6] De Oliveira GSJ, Castro-Alves LJ, Khan JH, McCarthy RJ (2013). Perioperative systemic magnesium to minimize postoperative pain: a meta-analysis of randomized controlled trials. Anesthesiology.

[7] Eide PK, Stubhaug A, Øye I (1995). 8 The NMDA-antagonist ketamine for prevention and treatment of acute and chronic post-operative pain. Baillieres Clin Anaesthesiol.

[8] Exadaktylos AK, Buggy DJ, Moriarty DC, Mascha E, Sessler DI (2006). Can anesthetic technique for primary breast cancer surgery affect recurrence or metastasis?. Anesthesiology.

[9] Ferlay J, Steliarova-Foucher E, Lortet-Tieulent J, Rosso S, Coebergh JWW, Comber H, Forman D, Bray F (2013). Cancer incidence and mortality patterns in Europe: estimates for 40 countries in 2012. Eur J Cancer.

[10] Fernández-Lao C, Cantarero-Villanueva I, Fernández-de-las-Peñas C, del-Moral-Ávila R, Menjón-Beltrán S, Arroyo-Morales M (2011). Widespread mechanical pain hypersensitivity as a sign of central sensitization after breast cancer surgery: comparison between mastectomy and lumpectomy. Pain Med.

[11] Fisher B, Bauer M, Margolese R, Poisson R, Pilch Y, Redmond C, Fisher E, Wolmark N, Deutsch M, Montague E, Saffer E, Wickerham L, Lerner H, Glass A, Shibata H, Deckers P, Ketcham A, Oishi R, Russell I (1985). Five-year results of a randomized clinical trial comparing total mastectomy and segmental mastectomy with or without radiation in the treatment of breast cancer. N Engl J Med.

[12] Fodale V, D’Arrigo MG, Triolo S, Mondello S, la Torre D (2014). Anesthetic techniques and cancer recurrence after surgery. ScientificWorldJournal.

[13] Forget P, Cata J (2017). Stable anesthesia with alternative to opioids: are ketamine and magnesium helpful in stabilizing hemodynamics during surgery? A systematic review and meta-analyses of randomized controlled trials. Best Pract Res Clin Anaesthesiol.

[14] Frauenknecht J, Kirkham KR, Jacot-Guillarmod A, Albrecht E (2019). Analgesic impact of intra-operative opioids vs. opioid-free anaesthesia: a systematic review and meta-analysis. Anaesthesia.

[15] Gan TJ, Diemunsch P, Habib AS, Kovac A, Kranke P, Meyer TA, Watcha M, Chung F, Angus S, Apfel CC, Bergese SD, Candiotti KA, Chan MT, Davis PJ, Hooper VD, Lagoo-Deenadayalan S, Myles P, Nezat G, Philip BK, Tramèr MR, Society for Ambulatory Anesthesia (2014). Consensus guidelines for the management of postoperative nausea and vomiting. Anesth Analg.

[16] Hontoir S, Saxena S, Gatto P, Khalife M, Ben Aziz AM, Paesmans M, Sosnowski M (2016). Opioid-free anesthesia: what about patient comfort? A prospective, randomized, controlled trial. Acta Anaesthesiol Belg.

[17] Jabbour HJ, Naccache NM, Jawish RJ (2014). Ketamine and magnesium association reduces morphine consumption after scoliosis surgery: prospective randomised double-blind study. Acta Anaesthesiol Scand.

[18] James MFM (2009). Magnesium: an emerging drug in anaesthesia. Br. J. Anaesth..

[19] Jolley S (2001). Managing post-operative nausea and vomiting. Nurs Stand.

[20] Kasai S, Ikeda K (2011). Pharmacogenomics of the human μ-opioid receptor. Pharmacogenomics.

[21] Kehlet H, Jensen TS, Woolf CJ, Centre M (2006). Persistent postsurgical pain: risk factors and prevention. Lancet.

[22] King CA, Perez-Alvarez IM, Bartholomew AJ, Bozzuto L, Griffith K, Sosin M, Thibodeau R, Gopwani S, Myers J, Fan KL, Tousimis EA (2020). Opioid-free anesthesia for patients undergoing mastectomy: a matched comparison. Breast J.

[23] Laskowski K, Stirling A, McKay WP, Lim HJ (2011). A systematic review of intravenous ketamine for postoperative analgesia. Can J Anesth.

[24] Lauridsen MC, Overgaard M, Overgaard J (2008). Shoulder disability and late symptoms following surgery for early breast cancer. Acta Oncol.

[25] Lee M, Silverman SM, Hansen H (2011). A comprehensive review of opioid-induced hyperalgesia. Pain Physician.

[26] Macintyre PE, Walker S, Power I, Schug SA (2006). Acute pain management: scientific evidence revisited. Br. J. Anaesth..

[27] Mansour MA, Mahmoud AAA, Geddawy M (2013). Nonopioid versus opioid based general anesthesia technique for bariatric surgery: a randomized double-blind study. Saudi J Anaesth.

[28] Marla S, Stallard S (2009). Systematic review of day surgery for breast cancer. Int J Surg.

[29] Massoth C, Schwellenbach J, Saadat-gilani K (2021). Impact of opioid-free anaesthesia on postoperative nausea, vomiting and pain after gynaecological laparoscopy - a randomised controlled trial. J Clin Anesth.

[30] Miaskowski C (2009). A review of the incidence, causes, consequences, and management of gastrointestinal effects associated with postoperative opioid administration. J perianesthesia Nurs Off J Am Soc PeriAnesthesia Nurses.

[31] Mulier J (2017). Opioid free general anesthesia: a paradigm shift?. Rev. Esp. Anestesiol. Reanim..

[32] Mulier JP (2019). Is opioid-free general anesthesia for breast and gynecological surgery a viable option?. Curr Opin Anaesthesiol.

[33] Oddby-Muhrbeck E, Eksborg S, Helander A, Bjellerup P, Lindahl S, Lonnqvist P (2005). Blood-borne factors possibly associated with post-operative nausea and vomiting: an explorative study in women after breast cancer surgery. Acta Anaesthesiol Scand.

[34] Parsa FD, Cheng J, Stephan B, Castel N, Kim L, Murariu D, Parsa AA (2017). Bilateral breast reduction without opioid analgesics: a comparative study. Aesthetic Surg J.

[35] Peltoniemi MA, Hagelberg NM, Olkkola KT, Saari TI (2016). Ketamine: a review of clinical pharmacokinetics and pharmacodynamics in anesthesia and pain therapy. Clin Pharmacokinet.

[36] Rafiq S, Steinbrüchel DA, Wanscher MJ, Andersen LW, Navne A, Lilleoer NB, Olsen PS (2014). Multimodal analgesia versus traditional opiate based analgesia after cardiac surgery, a randomized controlled trial. J Cardiothorac Surg.

[37] Salom A, Harkouk H, Fletcher D (2021) Opioid-free anesthesia benefit – risk balance : a systematic review and meta-analysis of randomized controlled trials. J Clin Med 10(10). 10.3390/jcm10102069 10.3390/jcm10102069 PMC8150912 34065937

[38] Samuels D, Abou-samra A, Dalvi P (2017). Journal of clinical anesthesia and opioid-free anesthesia results in reduced post-operative opioid consumption. J Clin Anesth Pain Med.

[39] Schnider TW, Minto CF, Gambus PL, Andresen C, Goodale DB, Shafer SL, Youngs EJ (1998). The influence of method of administration and covariates on the pharmacokinetics of propofol in adult volunteers. Anesthesiology.

[40] Schug SA, Palmer GM, Scott DA, Halliwell R, Trinca J (2016). Acute pain management: scientific evidence, fourth edition, 2015. Med J Aust.

[41] Sultana A, Torres D, Schumann R (2017). Special indications for opioid free anaesthesia and analgesia, patient and procedure related: including obesity, sleep apnoea, chronic obstructive pulmonary disease, complex regional pain syndromes, opioid addiction and cancer surgery. Best Pract Res Clin Anaesthesiol.

[42] Tinterri C, Gatzemeier W, Costa A, Gentilini MA, Zanini V, Regolo L, Pedrazzoli C, Rondini E, Amanti C, Gentile G, Taffurelli M, Fenaroli P, Tondini C, Sacchetto G, Sismondi P, Murgo R, Orlandi M, Cianchetti E, Andreoli C (2014). Breast-conservative surgery with and without radiotherapy in patients aged 55–75 years with early-stage breast cancer: a prospective, randomized, multicenter trial analysis after 108 months of median follow-up. Ann Surg Oncol.

[43] Tripathy S, Rath S, Agrawal S, Rao PB, Panda A, Mishra TS, Nayak S (2018). Opioid-free anesthesia for breast cancer surgery: an observational study. J Anaesthesiol Clin Pharmacol.

[44] Wesmiller SW, Bender CM, Conley YP, Bovbjerg DH, Ahrendt G, Bonaventura M, Sereika SM (2017). A prospective study of nausea and vomiting after breast cancer surgery. J perianesthesia Nurs Off J Am Soc PeriAnesthesia Nurses.

[45] Wesmiller SW, Sereika SM, Bender CM, Bovbjerg D, Ahrendt G, Bonaventura M, Conley YP (2017). Exploring the multifactorial nature of postoperative nausea and vomiting in women following surgery for breast cancer. Auton Neurosci.

[46] Ziemann-Gimmel P, Goldfarb AA, Koppman J, Marema RT (2014). Opioid-free total intravenous anaesthesia reduces postoperative nausea and vomiting in bariatric surgery beyond triple prophylaxis. Br J Anaesth.

